# The alternative coproporphyrinogen III oxidase (CgoN) catalyzes the oxygen-independent conversion of coproporphyrinogen III into coproporphyrin III

**DOI:** 10.3389/fmicb.2024.1378989

**Published:** 2024-03-13

**Authors:** Toni Mingers, Stefan Barthels, Violetta Mass, José Manuel Borrero-de Acuña, Rebekka Biedendieck, Ana Cooke, Tamara A. Dailey, Svetlana Gerdes, Wulf Blankenfeldt, Harry A. Dailey, Martin J. Warren, Martina Jahn, Dieter Jahn

**Affiliations:** ^1^Institute of Microbiology, University of Technical Engineering, Braunschweig, Germany; ^2^Pieris Pharmaceuticals GmbH, Hallbergmoos, Germany; ^3^Departamento de Microbiología, Facultad de Biología, Universidad de Sevilla, Sevilla, Spain; ^4^Department of Structure and Function of Proteins (SFPR), Helmholtz Centre for Infection Research (HZI), Institute for Biochemistry, Biotechnology and Bioinformatics, Braunschweig University of Technology, Braunschweig, Germany; ^5^School of Biosciences, University of Kent, Canterbury, United Kingdom; ^6^Quadram Institute Bioscience, Norwich, United Kingdom; ^7^School of Biological Sciences, University of Kent, Canterbury, United Kingdom; ^8^Department of Microbiology, University of Georgia, Athens, GA, United States; ^9^Svetalana Gerdes, Dupont Daniscao Research Center, Wilmington, DE, United States; ^10^Braunschweig Integrated Centre of Systems Biology, Braunschweig University of Technology, Braunschweig, Germany

**Keywords:** alternative heme biosynthesis, coproporphyrinogen III oxidase, Bacillaceae, *Priestia megaterium*, anaerobic metabolism

## Abstract

Nature utilizes three distinct pathways to synthesize the essential enzyme cofactor heme. The coproporphyrin III-dependent pathway, predominantly present in *Bacillaceae*, employs an oxygen-dependent coproporphyrinogen III oxidase (CgoX) that converts coproporphyrinogen III into coproporphyrin III. In this study, we report the bioinformatic-based identification of a gene called *ytpQ*, encoding a putative oxygen-independent counterpart, which we propose to term CgoN, from *Priestia* (*Bacillus*) *megaterium*. The recombinantly produced, purified, and monomeric YtpQ (CgoN) protein is shown to catalyze the oxygen-independent conversion of coproporphyrinogen III into coproporphyrin III. Minimal non-enzymatic conversion of coproporphyrinogen III was observed under the anaerobic test conditions employed in this study. FAD was identified as a cofactor, and menadione served as an artificial acceptor for the six abstracted electrons, with a *K_M_* value of 3.95 μmol/L and a *kcat* of 0.63 per min for the substrate. The resulting coproporphyrin III, in turn, acts as an effective substrate for the subsequent enzyme of the pathway, the coproporphyrin III ferrochelatase (CpfC). Under aerobic conditions, oxygen directly serves as an electron acceptor, but is replaced by the more efficient action of menadione. An AlphaFold2 model of the enzyme suggests that YtpQ adopts a compact triangular shape consisting of three domains. The N-terminal domain appears to be flexible with respect to the rest of the structure, potentially creating a ligand binding site that opens and closes during the catalytic cycle. A catalytic mechanism similar to the oxygen-independent protoporphyrinogen IX oxidase PgoH1 (HemG), based on the flavin-dependent abstraction of six electrons from coproporphyrinogen III and their potential quinone-dependent transfer to a membrane-localized electron transport chain, is proposed.

## Introduction

**Figure 1:**
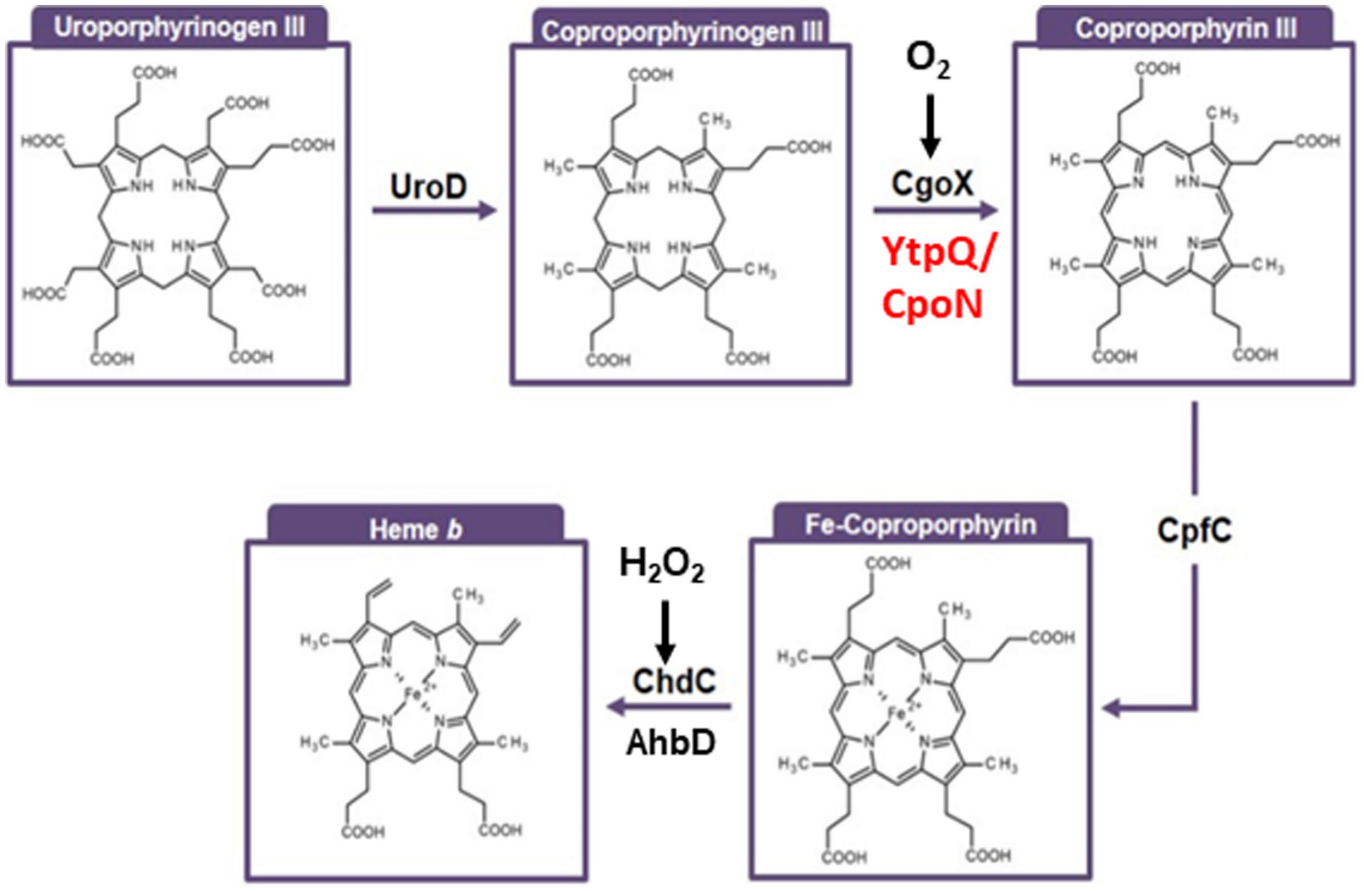
Pathway for the coproporphyrin-dependent heme *b* biosynthesis. This pathway is specific for Gram-positive *Bacillaceae*. First, decarboxylation catalyzed by UroD (uroporphyrinogen III decarboxylase) results in coproporphyrinogen III formation. Subsequent oxidation catalyzed by CgoX and CgoN (coproporphyrinogen III oxidases) leaves the name-giving coproporphyrin III intermediate. Next, iron is inserted by CpfC (coproporphyrin III ferrochelatase) to yield iron-coproporphyrin III (coproheme). A final decarboxylation is catalyzed by ChdC (coproheme decarboxylase) leading to the final product heme *b.*

Hemes are essential cofactors of multiple catalytically active enzymes and proteins of electron transport chains. They participate in the detection and the transport of various gases as components of sensors, regulators and hemoglobins (Dutt et al., 2022). Hemes are synthesized using multiple different pathways (Dailey et al., 2017; Jahn et al., 2021; Layer et al., 2022; Medlock and Dailey, 2022). Two different routes for the formation of the general heme precursor molecule 5-aminolevulinic acid are known (Heinemann et al., 2008; Layer, 2021). The central pathway from 5-aminolevulinic acid to the first ring-closed tetrapyrrole uroporphyrinogen III is conserved in all heme synthesizing organisms. From uroporphyrinogen III, most eukaryotic organisms and Gram-negative bacteria form coproporphyrinogen III, decarboxylate it to protoporphyrinogen IX prior oxidation to protoporphyrin IX with the final step involving insertion of iron for protoheme formation (Dailey et al., 2017). Oxygen-dependent and-independent alternatives exist for some enzymatic steps. Archaea, using what is believed to be a more primordial pathway, convert uroporphyrinogen III into siroheme which subsequently gets metabolized in a four-step oxygen-independent route towards protoheme (Bali et al., 2011; Layer, 2021). For many years it was assumed that *Bacillaceae* like other bacteria synthesize heme via the protoporphyrin-dependent pathway (Hansson and Hederstedt, 1992; Hippler et al., 1997; Homuth et al., 1999). However, with an increasing number of fully sequenced genomes available it became evident that most *Bacillaceae* do not encode for a gene for coproporphyrinogen decarboxylase (HemF, CgdC) or coproporphyrinogen dehydrogenase (HemN, CgdH) and hence should be unable to convert coproporphyrinogen III into protoporphyrinogen IX (Dailey et al., 2015). In 2010, it was shown that the *hemQ* gene, which is solely be found in *Bacillaceae* is essential for heme biosynthesis and does generally not coexist with *hemN* or *h*e*mF*. It was subsequently demonstrated that *hemQ* encodes for a coproheme decarboxylase (Dailey et al., 2010; Dailey and Gerdes, 2015). These findings led to a re-evaluation of biochemical, genetical and structural data from the past decades for the enzymes involved in heme biosynthesis in these bacteria. In 2015 a third heme *b* biosynthetic pathway was discovered (Figure 1; Lobo et al., 2015; Dailey et al., 2017). The newly-discovered coproporphyrin-dependent heme biosynthesis route branches at coproporphyrinogen III off the classical protoporphyrin-dependent path. The oxygen-dependent enzyme which catalyzes the six-electron oxidation of coproporphyrinogen III to coproporphyrin III is the *hemY-*encoded coproporphyrinogen III oxidase (CgoX). The *hemY*-type genes also encode the protoporphyrinogen oxidase (PgoX) of the classical pathway (Dailey et al., 2017). When first characterized in *Bacillus subtilis* as potential protoporphyrinogen IX oxidase, it was already noticed that HemY was also able to oxidize coproporphyrinogen III and that a *B. subtilis* Δ*hemY* deletion mutant accumulated coproporphyrin III (Hansson and Hederstedt, 1994). At that time, it was believed that coproporphyrin III represents a dead-end product of tetrapyrrole biosynthesis. Recent re-evaluation revealed that HemY from some Gram-positive bacteria showed predominantly coproporphyrinogen oxidase (CgoX) activity. *In vitro*, FAD serves as cofactor and the six abstracted electrons are transferred onto three oxygen molecules resulting in the formation of three hydrogen peroxide molecules as shown for the mammalian enzyme (Ferreira et al., 1988; Lobo et al., 2015). These biochemical data correlate with those of the highly related protoporpyhrinogen oxidase IX (PgoX) (Hansson et al., 1997; Dailey and Gerdes, 2015; Lobo et al., 2015). However, for the anaerobic growth of several *Bacillaceae* (Hoffmann et al., 1995) a second, oxygen-independent, yet unknown enzyme is required which is subject of this investigation. Next, iron gets inserted into coproporphyrin III yielding Fe-coproporphyrin III. This reaction is catalyzed by the *hemH* encoded coproporphyrin ferrochelatase (CpfC). A *Staphylococcus aureus* Δ*hemH* deletion mutant was observed to accumulate coproporphyrin III rather than protoporphyrin IX, yet giving a first indication that HemH from Gram-positive bacteria serves as a CpfC (Lobo et al., 2015). Similar to HemY, HemH-type enzymes from different organisms encode for two different enzymes, the protoporphyrin IX ferrochelatase PpfC and the coproporphyrin ferrochelatase CpfC. The last step of the coproporphyrin-dependent heme biosynthesis is the conversion of Fe-coproporphyrin III into heme *b*. This decarboxylation of ring A and B propionates to vinyl groups is catalyzed by the *hemQ* encoded coproheme decarboxylase (ChdC) (Dailey et al., 2010; Mayfield et al., 2013; Dailey and Gerdes, 2015; Hofbauer et al., 2015). Even though ChdC catalyzes the same reaction as the earlier described heme synthase AhbD (alternative heme biosynthesis enzyme D of archaea) (Kuhner et al., 2016), a structural similarity of both enzymes was not found. HemQ (ChdC) was shown to be oxygen-dependent and requires H_2_O_2_ for its reaction. Again, a second, yet unknown oxygen-independent enzyme is required for the anaerobic growth of the bacteria. Here we describe the identification and biochemical characterization of the oxygen-independent coproporphyrinogen III oxidase YtpQ (CgoN).

## Materials and methods

### Identification and phylogenetic investigation of YtpQ as a putative missing heme synthesizing enzyme in *Bacillaceae*

To identify genes that might encode a missing heme synthesis enzyme(s) we employed the SEED data base. The overall bioinformatics approach for the identification of novel coproporphyrinogen III utilizing enzymes in bacteria using the SEED database was outlined before (Overbeek et al., 2005; Boynton et al., 2011; Dailey et al., 2015). This approach was previously successful in identifying HemQ/ChdC as an enzyme essential for heme synthesis in the Actinobacteria and Firmicutes (Dailey et al., 2010, 2015). In searching for a putative anaerobic coproporphyrinogen III oxidase three criteria were employed: (1) Members of a candidate protein family should be present only in genomes containing other genes of the heme branch of tetrapyrrole biosynthesis and they should not occur in organisms incapable of heme biosynthesis. (2) Co-localization with other genes of heme biosynthesis was considered, but not employed as a strict requirement. (3) Gene expression patterns, when available, were used to limit the list of potential candidates to only those hypothetical genes which display “expression patterns” similar to that of known tetrapyrrole biosynthesis genes. This analysis yielded a member of an annotated hypothetical ORF (DUF1444 protein superfamily). In *B. subtilis* this is annotated as *ytpQ* and in *S. aureus* as SAV1743.

For phylogenetic analyses, the protein YtpQ (WP_013085235.1) of *P. megaterium* was blasted using the NCBI protein database. Proteins with at least 60% amino acid sequence identity and a coverage over 70% were considered for initial analyses. Below these thresholds several other proteins were included as they were found in organisms encompassing potential outliers, including Gram-negative bacteria or bacteria not expected to have the YtpQ protein given their biochemical background. Subsequently, protein sequences representative for a distinct group of bacteria were retrieved from the NCBI Database for phylogenetic analyses. Corresponding FASTA files were transferred to MEGA 11 (Tamura et al., 2021) for alignment using the ClustalW algorithm. The arising files were exported (as .mega) to perform evolutionary analyses employing the Maximum Likelihood method and a JTT matrix-based model (Jones et al., 1992) (500 bootstrap). The generated file (.mtsx) was imported as a Newick file into the iTOL software (Letunic and Bork, 2019) for customization of the final tree display.

### Recombinant production and purification of *Priestia megaterium* YtpQ

The synthetic *P. megaterium ytpQ* gene (Gene ID: 64145469, for CE057_RS06095, a DUF1444 domain-containing protein, protein ID WP_013085235.1) modified to *E. coli* K12 codon usage using the codon adaption tool JCat^1^ (Grote et al., 2005) was purchased from New England Biolabs GmbH (Frankfurt a. M., Germany). The gene was inserted via its synthetic linkers into the *Bam*HI and *Not*I restriction sites of the pGEX-6p-1 expression vector creating pGEX-6P-1-*P. megaterium ytpQ* encoding an N-terminal glutathione S-transferasetag (GST-tag) with a PreScission™ protease cleavage site, an isopropyl-β-D-thiogalactosidase (IPTG) inducible *lac* promotor and an ampicillin resistance cassette (GE Healthcare™ GmbH, Freiburg, Germany). The integrity of the final vector was verified by complete DNA sequencing (Eurofins Genomics, Ebersberg, Germany). For protein production and purification, the vector pGEX-6p-1_*P. megaterium ytpQ* was introduced into *E. coli* BL21(DE3). Cultures of 2 L LB-broth containing 100 mg/L ampicillin were inoculated with an *E. coli* overnight cultures to a starting OD_578_ of 0.05 and grown aerobically with an agitation of 200 rpm at 37°C. The *ytpQ* gene expression was induced during late exponential growth phase at OD_578_ of 0.6 by addition of 125 μM IPTG. The culture was continuously incubated for 20 h at 17°C and 180 rpm. The following cell disruption and protein purification processes were conducted under strictly anaerobic conditions at 4°C. Then, the overnight cultures were transferred to anaerobic 1 L centrifuge tubes, and cells were harvested by centrifugation at 3500 × *g* for 20 min at 4°C. The harvested cells were re-suspended in 8 mL ice-cold buffer I (40 mM HEPES, pH 7.2, 5% (v/v) glycerol, 1 mM dithiothreitol (DTT)) and disrupted by a double passage through a French Press (Thermo Fisher Scientific Inc., Waltham, USA) at 132,379, 200 Pa (1323.792 bar, 19,200 psi). In order to remove cell debris, the sample was centrifuged for 60 min at 25,000 × *g* and 4°C. All following procedures were performed in an anaerobic chamber under strict anaerobic conditions. Subsequently, the soluble protein fraction was applied onto an Econo-Pac® chromatography column loaded with 5 mL anaerobic Protino® glutathione agarose 4B (Machery-Nagel KG, Düren, Germany) that was prior equilibrated with 10 column volumes buffer I. The cell-free extract was mixed with the column material and incubated for 2 h at 4°C to allow binding of the YtpQ-GST recombinant protein to the matrix. Afterwards, the flow-through was collected and the column was washed with 6 column volumes of buffer I. YtpQ was eluted from the column by cleavage of the GST-tag overnight using 800 U PreScission™ protease (GE Healthcare™ GmbH, Freiburg, Germany) diluted in 6 mL anaerobic buffer III (10 mM Na_2_HPO_4_, 1.8 mM KH_2_PO_4_, pH 7.4, 5% (v/v) glycerol, 1 mM DTT, 140 mM NaCl, 2.7 mM KCl). Next, the GST-tag was removed from the column material by rinsing with 15 mL buffer II (buffer I with 10 mM glutathione). Protein concentrations were determined using Bradford reagent (Sigma-Aldrich Chemie, Taufkirchen, Germany) according to manufacturer’s instructions. The protein composition of fractions of interest was visualized via a SDS-PAGE.

### Gel permeation chromatography

A Superdex® 200 HR 10/30 column run on an Äkta purifier system (GE Healthcare™ GmbH, Freiburg, Germany) was equilibrated under anaerobic condition in an anaerobic chamber with protein buffer I and calibrated using the Gel Filtration Molecular Weight Markers Kit MWGF200 (Sigma Aldrich, Chemie, Taufkirchen, Germany) composed of cytochrome c (*M*_r_ = 12,400), carbonic anhydrase (*M*_r_ = 29,000), bovine serum albumin (*M*_r_ = 66,000), alcohol dehydrogenase (*M*_r_ = 150,000), β-amylase (*M*_r_ = 200,000), and apoferritin (*M*_r_ = 400,000) according to the manufacturer’s instructions. Purified protein was filtered through a 0.22 μm syringe filter (Sarstedt, Germany) before the 2 mg/ml protein solution in buffer I was chromatographed at a flow rate of 0.5 mL/min. The procedure was monitored via an UV detector at 280 nm.

### Coproporphyrinogen III oxidase activity assay

In order to identify YtpQ as oxygen-independent coproporphyrinogen III oxidase (CgoN) catalyzing the conversion of coproporphyrinogen III into coproporphyrin III an anaerobic enzyme activity assay was established. All experiments, if not indicated otherwise, were done under light protected and anoxic conditions using oxygen-depleted solutions and materials in an anaerobic chamber (Coy Lab products, Grass Lake, MI, USA). In contrast to the substrate coproporphyrinogen III the product of the reaction coproporphyrin III is fluorescent. Thus, its formation was followed by fluorescence measurements with an excitation at 409 nm and emission measurements between 580 and 650 nm using a FP8000 fluorimeter (JASCO, Pfungstadt, Germany) in a high sensitivity mode. The excitation and emission bandwidths were 2.4 nm. The samples were tested in 115F microcuvettes under anaerobic conditions closed with a plug (Hellma Analytics, Mullheim, Germany) during the measurement to avoid oxidation. Spectra were finally analyzed using the JASCO software Spectra Manager™. In the 500 μl standard CgoN testing setup, first buffer IV (buffer I including 0.02% Tween 80 was pre-warmed to 30°C). One μM freshly purified YtpQ and the respective indicated additives were added in the concentration to be tested. The reaction was started by addition of a solution of the also freshly prepared substrate coproporphyrinogen III to a final concentration of 10 μM, incubated at 30°C and 200 rpm for indicated time points, usually for 120 min. All fluorescence measurements were done at least in triplicates. Emission peak values at 614 nm were extracted and the respective non-enzyme control values subtracted from the enzyme probe values. Calibration curves were generated for each coproporphyrinogen III batch using identical conditions to the CgoN activity measurements.

### *Priestia megaterium* CpfC (HemH) recombinant production, purification and testing in combination with CgoN

In order to test if the product of the recombinant CgoN catalysis is an efficient substrate for the subsequent enzyme coproporphyrin ferrochelatase (CpfC, HemH), the CpfC protein from *P. megaterium* was recombinantly produced in. *E. coli* and chromatographically purified. For this purpose, the corresponding *hemH* gene (locus tag QY062_23700) was PCR amplified from genomic *P. megaterium* DNA using the primers BmhemH.for (5´ CCCGGGTCGACTCA TGAGTAAAAAAGTAATGGGATTACT 3′) and BmhemH.rev (5’ TTATGCGGCCGCTCTTAACGGGCAATCTGTTTTAA 3′). The resulting PCR product was cut with *Sal*I and *Not*I and ligated into the appropriately digested vector pGEX-6P-1 resulting in pGEX-6P-1-*hemH-Bacillus megaterium* encoding an N-terminal glutathione S-transferasetag (GST-tag) with a PreScission™ protease cleavage site, an isopropyl-β-D-thiogalactosidase (IPTG) inducible *lac* promotor and an ampicillin resistance cassette (GE Healthcare™ GmbH, Freiburg, Germany). Overall integrity of the vector was ensured by complete DNA sequencing. For protein production and purification, the vector pGEX-6P-1-*hemH-Bacillus megaterium* was transformed into *E. coli* BL21(DE3). Cultures of 4 L LB-broth containing 100 mg/L ampicillin were inoculated with overnight cultures to a starting OD_578_ of 0.1 and grown aerobically with an agitation of 200 rpm at 37°C. The *hemH* gene expression was induced during late exponential growth phase at OD_578_ of 0.4 by addition of 250 μM IPTG. The culture was continuously incubated for 14 h at 17°C and 200 rpm. Cell breakage, cell debris removal and chromatographic purification including proteolytic release of *P. megaterium* HemH (CpfC) (GenBank accession WPL43387) from the column bound GST-HemH fusion protein (35 kDa CpfC +26 kDa GST = 61 kDa YtpQ-GST) was performed as outlined above for the YtpQ (CgoN) protein. SDS PAGE analysis showed a major single band with an approximate M_r_ of 40.000 ± 3,000 (calculated molecular weight 35,199 Da) in the final elution fraction. As observed by our groups already for the *B. subtilis* HemH, *P. megaterium* HemH appeared with a little bit larger M_r_ during SDS PAGE analysis, probably due to its elongated asymmetric structure. Nevertheless, protein identity and integrity were confirmed by mass spectrometry. Overall, 9 ± 2.1 mg/L HemH protein per L cell culture were obtained. Next, the enzyme was tested in combination with purified *P. megaterium* CgoN in a standard CgoN enzyme assay as outlined above. One μM freshly purified CgoN, 1 μM freshly purified CpfC, 5 μM menadione, 3 μM FAD, and 10 μM (NH_4_)_2_Fe(SO_4_)_2_ were mixed in prewarmed buffer IV. The 300 μL reaction was started by the addition of 10 μM freshly prepared coproporphyrinogen III and was incubated at 30°C and 200 rpm for 120 min. Subsequently, the reaction was stopped by the addition of 10 μL concentrated HCl, then 600 μL acetone/HCl (97.5%/2.5% v/v) were added and the mixed solution stored for 10 min on ice. Precipitated proteins were harvested via centrifugation for 20 min at 12,000 × *g* at 4°C. The sterile filtered supernatant (200 μL) was subjected to HPLC separation using a Equisil BDS C_18_ column (5 μm particle diameter, 250 mm × 4.6 mm size, Dr. Maisch HPLC GmbH, Ammerbuch-Entringen, Germany) at a flow rate of 0.5 ml/min on Jasco HPLC system (LG1580) using detectors (FP 1520 and MD 1515) with 1 M ammonium acetate pH 5,2 as solvent A, methanol as solvent B, and acetonitrile as solvent C. Samples were resolved after injection via a gradient of solvent A and B, while solvent C remained at 10% throughout the whole procedure. The gradient was during min 1–25 min from 60% solvent A and 30% solvent B to 15% A and 75% B. During min 25 to min 30 it went to 0% A and 90% B, and stayed until 50 min. The column was calibrated with coproporphyrinogen III (27, 60 min) and Fe-coproporphyrinogen III (coproheme, 27, 75 min).

### Identification of the product of CgoN (YtpQ) catalysis using mass spectrometry and absorbance spectroscopy

One μM freshly purified CgoN, 5 μM menadione, 3 μM FAD were mixed in prewarmed 300 μL buffer IV under anaerobic conditions. After addition of 10 μM freshly prepared coproporphyrinogen III and incubation at 30°C and 200 rpm for 120 min, the reaction was stopped by the addition of 10 μL concentrated HCl. Proteins were precipitated using 600 μL acetone/HCl (97.5%/2.5% v/v) for 10 min on ice and were harvested via centrifugation for 20 min at 12,000 x g at 4°C. The sterile filtered supernatant (200 μl) was lyophilized for the transfer to cooperation partners. Lyophilized samples were there resuspended in 1 ml 100 mM HCl and resolved on an ACE 5AQ column (2.1 × 150 mm; Advanced Chromotography Technologies Ltd., Aberdeen, UK) attached to an Agilent 1100 series HPLC system equipped with a diode array detector and coupled to a microTOF-Q mass spectrometer (Bruker, Coventry, UK). The column was treated with a gradient at a flow rate of 0.2 mL min^−1^. Solvent A was 0.1% (v/v) TFA and solvent B acetonitrile (100%). After injection, solvent B was increased during 0 to 10 min from 20 to 50%, during10 to 30 min from 50 to 100%, and remained for 10 min at 100%, before it was finally decreased to 20% again for another 5 min.

## Results

### Bioinformatic-based identification of *ytpQ* as gene for a potential alternative coproporphyrinogen III oxidase

**Figure 2:**
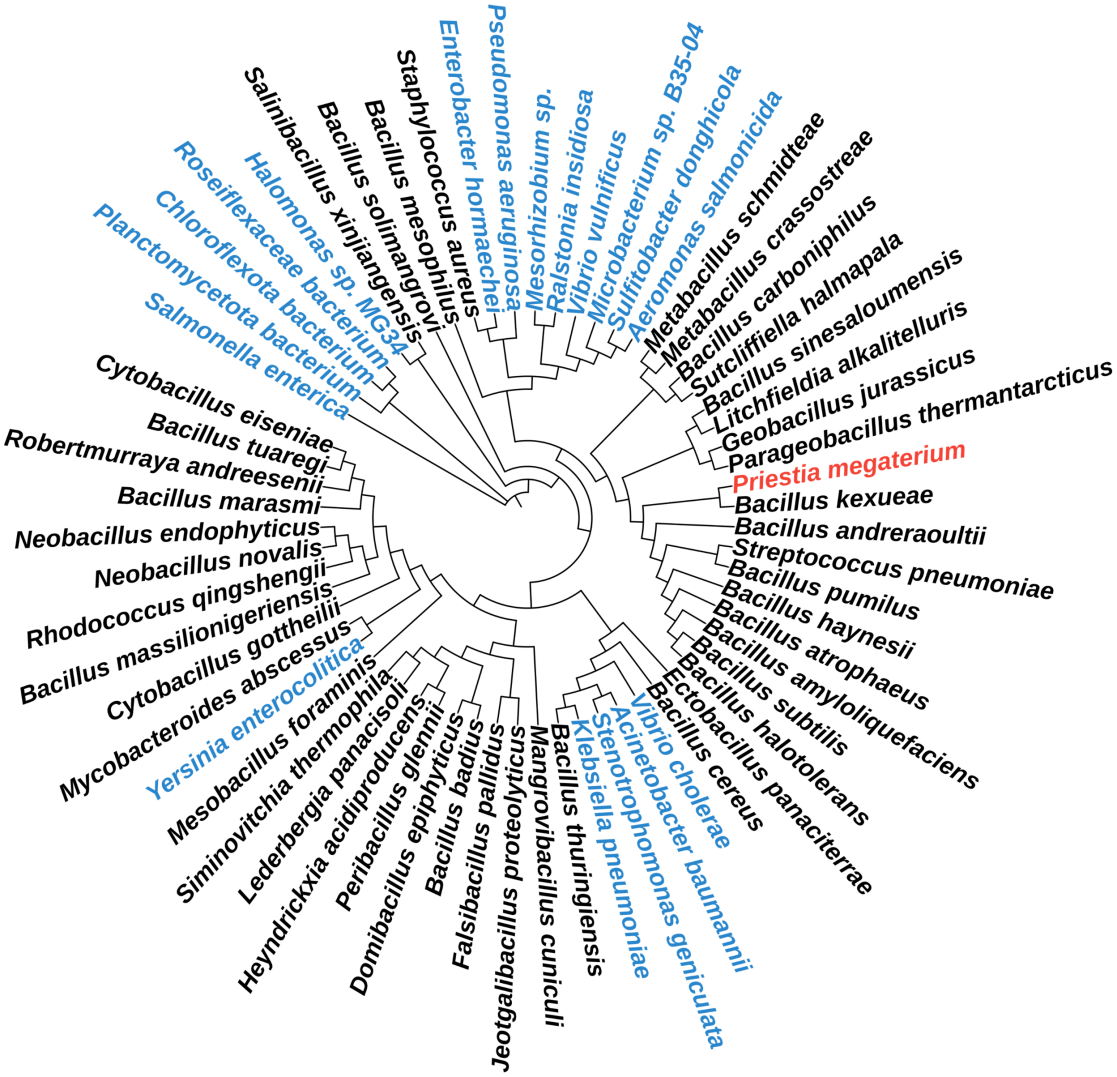
Phylogenetic distribution of YtpQ and YptQ-like proteins across different bacteria. The phylogenetic tree was built using Mega 11-based maximum likelihood analyses with 500 bootstrap replicates for the YtpQ protein. The iTOL software was employed for tree display customization. Proteins belonging to Gram-negative bacterial strains are shown in blue. The query strain protein sequence is highlighted in red.

An examination of nearly 1,000 genomes was conducted to identify the colocalization of genes of unknown function alongside genes encoding enzymes of know function (*hemY*/*cgoX*, *hemH*/*cpfC*, *hemQ*/*chdC*) of the heme biosynthetic pathway in Actinobacteria/*Firmicutes*, utilizing the SEED database. This investigation revealed the *ytpQ* gene as a promising candidate, potentially encoding an alternative coproporphyrinogen III oxidase (CgoN), as described previously (Hansson et al., 1997). Subsequently, we examined the phylogenetic distribution of the identified YtpQ (CgoN) protein through the NCBI protein database. It was observed that approximately 98% of bacterial strains in the NCBI database harboring this protein exhibited typical length coverage of 98%, and their amino acid sequences showed identities ranging from 54 to 100%. Notably, these strains were predominantly classified under the *Bacilli* class and belonged to the *Bacillaceae* family (Figure 2). The protein was not found in other classes within the phylum *Firmicutes*, such as *Haloanaerobiales*, *Negativicutes*, *Thermoanaerobacteriales*, *Erysipelotrichia* or *Tenericutes*. In the *Clostridia* class, only three strains (*Eubacteriales* sp., *Butyricicoccus* sp. 1Xd8-22, *Lacrimispora indolis*) were identified that contained a YtpQ orthologue. Similarly, for the *Actinobacteria* phylum, only a limited number of YtpQ (CgoN)-containing strains were discovered. Exhibiting a length coverage of 98%, and amino acid sequence identities of 42–65%. These included three strains of *Mycobacteroides abscessus sub*sp. *abscessus* (accession No. SLL35700.1, SHO03354.1, SLB71249.1), one *Streptomyces* strain (sp. ISL-14, MBT2670766) and *Rhodococcus gingshengii* (TDL77636.1).

Surprisingly, a few Gram-negative bacterial strains were also identified. It is noteworthy, in this context, that the genomes of many of these species often encompass several hundred to several thousand strains. YtpQ proteins, featuring a length coverage of 94 to 98%, and amino acid identities of 53 to 66%, were observed in individual strains of *Yersinia enterocolitica* (HEO8420061.1), *Vibrio vulnificus* (TDL88440.1), *Halomonas* sp. *MG34* (NAP00950.1), *Ralstonia insidiosa* (MBA9874239.1), *Ralstonia pickettii* (MBN6206906.1), *Sulfitobacter donghicola* (QAV32039.1), *Stenotrophomonas* sp. 1198 (MDH8707271.1), *E. coli* (MCZ5875759.1), and two strains of *Klebsiella pneumoniae* (OON70437.1, PCO21711.1). Furthermore, YtpQ (CgoN)-like proteins were identified in a subset of Gram-negative bacterial strains, which can be classified into two types. The first type consisted of bacteria featuring a YtpQ (CgoN) type protein with a relatively short length coverage ranging from 34–77%. These proteins exhibited a strong amino acid identity of 47–60%. Notable examples include single strains of *Stenotrophomonas peniculata* (MCR1808434.1), *Salmonella enterica sero*var *Enteritidis* str. P125109 (HAE0521353.1), *Pseudomonas aeruginosa* (MUK58307.1), *Vibrio cholerae* O1 (MBU5908558.1), and various *Acinetobacter baumannii* strains (MCP8609383.1, MCP8622039). The second type was characterized by a longer length coverage ranging from of 71 to 85%, but with a lower amino acid identity of around 25%. This type included *Chloroflexi* bacterium SZAS-1 and related strains (MBS1965690.1, NWG21152.1, KAB814911.1), as well as diverse *Roseoflexus* sp. (MCS6939962.1, MCS6839983.1). Their relations are depicted in the phylogenetic tree shown in Figure 2. While a few bacterial strains carrying YtpQ (CgoN) proteins were found in multiple bacterial phyla, it is noteworthy that over 98% of the YtpQ (CgoN) proteins were identified in the *Bacillaceae* family.

### Recombinant production and purification of *Priestia megaterium* YtpQ (CgoN)

**Figure 3:**
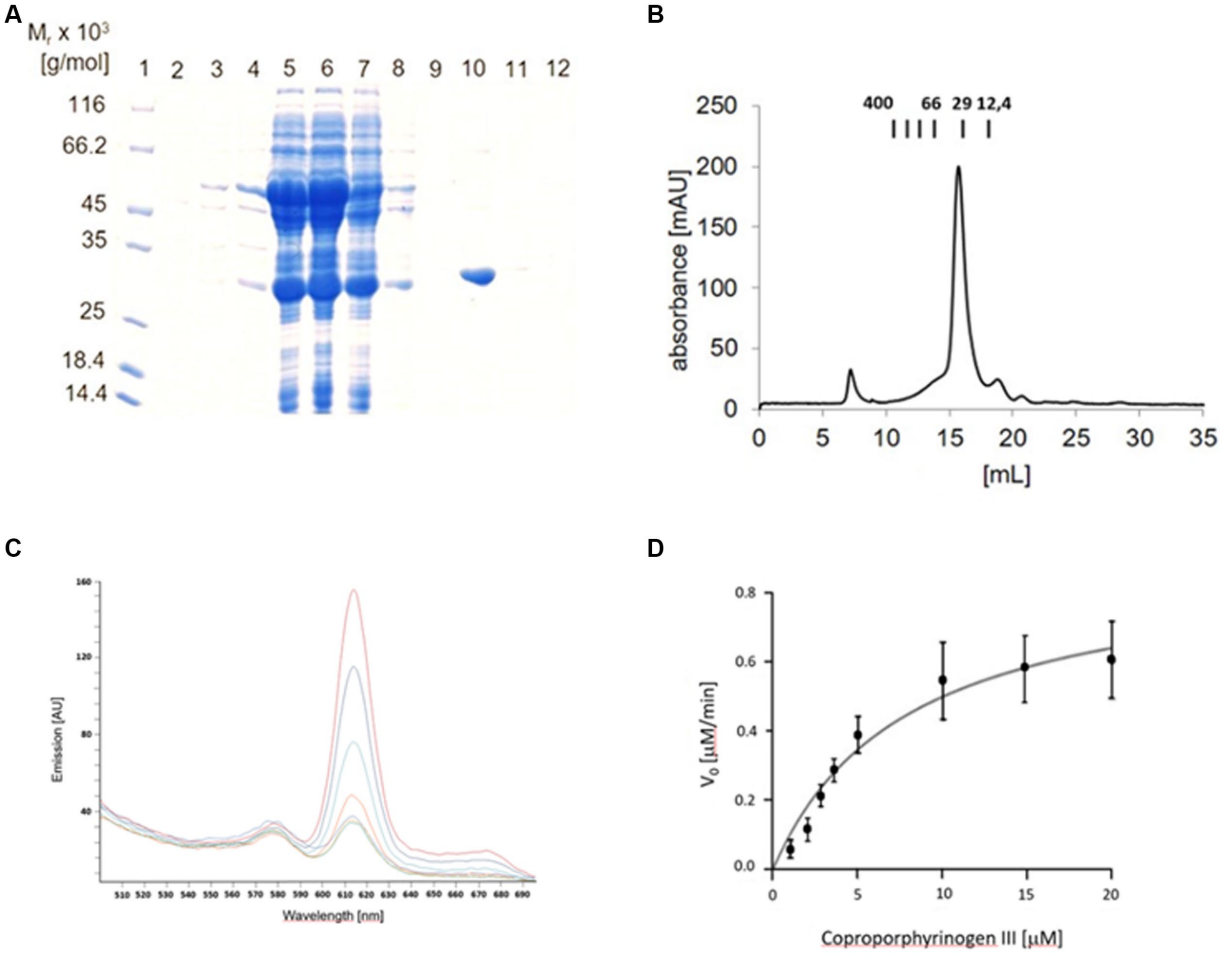
**(A)** SDS gel of the production and purification of *P. megaterium* YptQ (CgoN). Recombinant *P. megaterium* YptQ (CgoN) was produced in *E. coli* BL 21 as outlined in Material and Methods. The image shows an InstantBlue™ stained 12% SDS polyacrylamide gel after electrophoresis. Pierce™ unstained molecular weight marker protein ranging M_r_ from 14,000 to 116,000 are shown in lane 1. Further, total cellular extracts before IPTG induction (lane 2); 2 h (lane 3) and 20 h (lane 4) after the addition of 200 μM IPTG-are next; followed by the soluble (lane 5) and insoluble fraction of the extract (lane 6) after ultracentrifugation. The flow through of the glutathione agarose (lane 7), washing fraction 1 (lane 8); washing fraction 4 (lane 9), elution fraction 1 (lane 10) and fraction 2 (lane 11) after PreScission™ protease cleavage, and the final GST-tag elution fraction (lane 12) are concluding the analysis. Fraction 10 contains a single YptQ (CgoN) band with a M_r_ of 30,000 ± 3,000, nicely corresponding to the calculated molecular weight of the protein of 30,781 Da. **(B)** Native molecular weight and oligomerization state of *P. megaterium* YptQ determined via gel permeation chromatography. Analytical gel permeation chromatography of freshly purified recombinant *P. megaterium* YtpQ (CgoN) was performed using a 24 mL Superdex® 200 10/30 column on an Äkta purifier system under anaerobic conditions. Protein elution was followed by absorbance measurements at 280 nm. The column was equilibrated using the Gel Filtration Molecular Weight Markers Kit MWGF200 (Sigma Aldrich, Germany) composed of cytochrome *c* (*M*_r_ = 12,400), carbonic anhydrase (*M*_r_ = 29,000), bovine serum albumin (*M*_r_ = 66,000), alcohol dehydrogenase (*M*_r_ = 150,000), β-amylase (*M*_r_ = 200,000), and apoferritin (*M*_r_ = 400,000). Their elution position is given in *M*_r_ (x1,000) on the top of the figure. The chromatogram shows one major peak of *M*_r_ = 35,000 ± 5,000, indicating a monomeric YtpQ (CgoN) protein. **(C)** Time-resolved coproporphyrinogen III to coproporphyrin III conversion by *P. megaterium* YtpQ in the presence of FAD and menadione. Standard anaerobic YtpQ (CgoN) assays including 1 μM purified, recombinant YtpQ (CgoN), 10 μM coproporphyrinogen III, 3 μM FAD and 5 μM menadione were incubated at 30°C and 200 rpm. Samples were taken at different time points and analyzed by fluorescence spectroscopy with an excitation wavelength of 409 nm and emission measurements from 510 nm to 690 nm at 0 min (cyan), 5 min (turquoise), 10 min (yellow), 20 min (orange), 30 min (light blue), 45 min (dark blue), and 60 min (brown). **(D)** Kinetic analysis of coproporphyrinogen III oxidase activity of purified, recombinant *P. megaterium* YtpQ (CgoN). Anaerobic standard activity assays were conducted with 1 μM purified YtpQ (CgoN), 3 μM FAD and 5 μM menadione, and 1–20 μM of the substrate coproporphyrinogen III. A graphic representation of the initial velocity v_0_ of coproporphyrin III formation by the enzyme as a function of substrate concentration is shown. A *K*_M_ value of 3.95 μmol/L and a *k_cat_* of 0.63 min^−1^ were deduced.

Initially, the *ytpQ* gene of *Bacillus subtilis* was investigated. However, attempts to produce recombinant *B. subtilis* YtpQ (NCBI reference sequence WP_080332230.1) in *E. coli* resulted in the generation of a highly unstable protein that was prone to rapid degradation. As a result, we transitioned to a study of the *Priestia* (formerly *Bacillus*) *megaterium* gene. To enhance efficient recombinant protein production in *E. coli*, a synthetic codon-adapted *P. megaterium ytpQ* gene (Gene ID: 64145469) was utilized. This gene was fused with DNA encoding a N-terminal glutathione S-transferase (GST) tag, featuring a PreScission™ protease cleavage site. The YtpQ-GST fusion protein was subject to affinity chromatography on a glutathione Sepharose column, followed by PreScission™ protease-mediated release of YtpQ from the YtpQ-GST fusion protein (calculated molecular weight 57 kDa) under anaerobic conditions, yielding a protein with a molecular weight of 30 ± 3 kDa. The exact calculated molecular weight of YtpQ, which is 30,781 Da, aligns well with the size of the most predominant band observed in lane 10 of the SDS PAGE in Figure 3A. Subsequent mass spectrometry analysis of the protein in fraction 10 confirmed its identity as YtpQ. The average amount of purified recombinant protein obtained throughout this study was 7.3 ± 1.7 mg per L of *E. coli* culture.

### *Priestia megaterium* YtpQ (CgoN) is a monomeric protein

Following the successful production and purification of *P. megaterium* YptQ, the oligomeric state was determined using gel permeation chromatography (GPC). In this procedure, freshly anaerobically purified YtpQ was applied to 24 mL Superdex® 200 10/30 column and proteins eluting from the column were followed monitored spectroscopically at a wavelength of 280 nm. Figure 3B illustrates a prominent, well-defined absorption peak at approximately 16 mL, corresponding to a molecular weight of 35 ± 5 kDa. This observation led to the conclusion that the native protein adopts a monomeric structure.

### *Priestia megaterium* YtpQ (CgoN) is an oxygen-independent coproporphyrinogen III oxidase converting coproporphyrinogen III into coproporphyrin III

An anaerobic *in vitro* assay based on fluorescence measurements of the product coproporphyrin III was established and optimized. In this assay, 1 μM of anaerobically purified YtpQ protein was initially incubated with 10 μM coproporphyrin III under anaerobic conditions, and the sample was analyzed at different time points using fluorescence spectroscopy (excitation wavelength of 409 nm, emission wavelength of 615 nm). A critical aspect of this assay is the autocatalytic, non-enzymatic conversion of coproporphyrinogen III to coproporphyrin III, a process typically requiring oxygen. As indicated in Table 1, minimal non-enzymatic conversion (3 ± 1 arbitrary units = AU) of coproporphyrinogen III into coproporphyrin III was observed in the presence of bovine serum albumin as a control protein. However, when anaerobically purified YtpQ was present, a low but significant enzyme-catalyzed activity (15 ± 2 AU) was detected (Table 1). These measurements may have been influenced by residual amounts of potential cofactors bound to the recombinant enzyme. Subsequently, our focus shifted to identifying these cofactors.

**Table 1:** Parameters influencing YtpQ (CgoN) activity.

Sample	1	2	3	4	5	6	7	8	9	10	11	12	13
Enzyme activity (AU)	(3 ± 1)	15 ± 2	41 ± 4	161 ± 15	17 ± 2	16 ± 2	15 ± 2	17 ± 2	14 ± 2	157 ± 17	(13 ± 3)	50 ± 5	141 ± 19
YptQ (CgoN)	−	+	+	+	+	+	+	+	+	+	−	+	+
Coprogen	+	+	+	+	+	+	+	+	+	+	+	+	+
FMN	−	−	−	−	+	−	−	−	−	−	−	−	−
Hemin	−	−	−	−	−	+	−	−	−	−	−	−	−
FAD	+	−	+	+	−	−	−	−	−	+	+	+	+
EDTA/EGTA	−	−	−	−	−	−	−	−	−	+	−	−	−
Ubiquinone	−	−	−	−	−	−	+	−	−	−	−	−	−
Menaquinone	−	−	−	−	−	−	−	+	−	−	−	−	−
Phylloquinone	−	−	−	−	−	−	−	−	+	−	−	−	−
Menadione	+	−	−	+	−	−	−	−	−	+	−	−	+
Oxygen (Air)	−	−	−	−	−	−	−	−	−	−	+	+	+

The standard 120 min anaerobic CgoN assay is described in Material and Methods. The listed 500 μl assays included, where indicated, 1 μM freshly purified YtpQ, 10 μM coproporphyrinogen III, 3 μM FAD, 3 μM FMN, 3 μM heme, a mixture of 5 μM EDTA and EGTA, 5 μM ubiquinone, 5 μM menaquinone, 5 μM phylloquinone, or 5 μM menadione, respectively. Aerobic assays were performed in the presence of oxygen (air), where indicated. The product coproporphyrin III was detected by fluorescence measurements with an excitation at 409 nm and emission measurements at 614 nm and expressed as arbitrary units (AU). In calibration curves the coproporphyrinogen III substrate concentration of 10 μM accounted for approximately 250 AU. The background resulting from chemical conversion of coproporphyrinogen III to coproporphyrin III (anaerobic 3 ± 1 AU, aerobic, 13 ± 3 AU) was deducted from the obtained values.

### FAD is the cofactor and menadione the artificial electron acceptor of *Priestia megaterium* YtpQ (CgoN)

HemG (PgdH1) and HemY (CgoX, PgoX) are enzymes responsible for the oxygen-dependent and independent abstraction of six electrons from coproporphyrinogen III and protoporphyrinogen IX, respectively. In both cases, these processes require flavin cofactors. Additionally, HemG (PgdH1) couples the electron abstraction to the electron transport chain through quinone, functioning under both aerobic and anaerobic conditions. Conversely, the oxygen-dependent HemY (CgoX, PgoX) produces H_2_O_2_ as a byproduct (Boynton et al., 2009; Möbius et al., 2010). Given these insights, our investigation focused on assessing the potential stimulation of YtpQ activity by flavins and heme, serving as alternative electron carrier systems under anaerobic conditions. As indicated in Table 1, the addition of 3 μM FAD to 1 μM purified YtpQ significantly increased enzyme activity from 15 ± 2 AU to 41 ± 15 AU, whereas the addition of 3 μM heme and 3 μM FMN showed no discernable effect. Consequently, FAD was identified as the electron-transferring cofactor of YtpQ. Subsequently, we assessed the impact of various quinones (ubiquinone, menaquinone, phylloquinone, menadione) by adding 5 μM of each to 1 μM YtpQ as potential electron acceptors. As shown in Table 1, only the combination of 3 μM FAD with 5 μM menadione resulted in a notable increase in enzyme activity, from 15 ± 2 AU to 161 ± 15 AU. To eliminate the influence of metals on catalysis, the enzyme was treated with a combination of EDTA and EGTA (5 μM each), yet no significant effect was observed (Table 1). Similarly, the addition of metals did not impact YtpQ catalysis. No significant metal content was detected by a commercial atomic absorption-based metal analysis of the recombinant purified protein.

Consequently, we conducted a time-dependent assessment of coproporphyrinogen III to coproporphyrin III conversion in the presence of FAD and menadione (Figure 3C). The fluorescence emission spectrum exhibited a progressive increase in the characteristic coproporphyrin III peak, prominently observed at 615 nm. After 2 h of anaerobic catalysis, a peak intensity of about 160 AU was reached, in good agreement with the previous tests detailed in Table 1. The initial coproporphyrinogen preparation included some residual coproporphyrin III, identified by an initial peak at 615 nm. In the control reaction with BSA as the control protein and without YtpQ, this residual amount remained unchanged under anaerobic conditions, serving as a consistent background value subtracted from the measured values. The substrate concentration of coproporphyrinogen III used was 10 μM, corresponding to approximately 250 AU. Consequently, the enzyme reaction stopped at about 120 min with a conversion rate of 64%. By employing this anaerobic assay we measured a *K_M_* value of 3.95 μmol/L and a *k_cat_* of 0.63 min^−1^ for the recombinant, purified YptQ (Figure 3D).

### Identification of the enzyme reaction product coproporphyrin III by mass spectrometry and its conversion by the subsequent enzyme of the pathway (CpfC)

**Figure 4:**
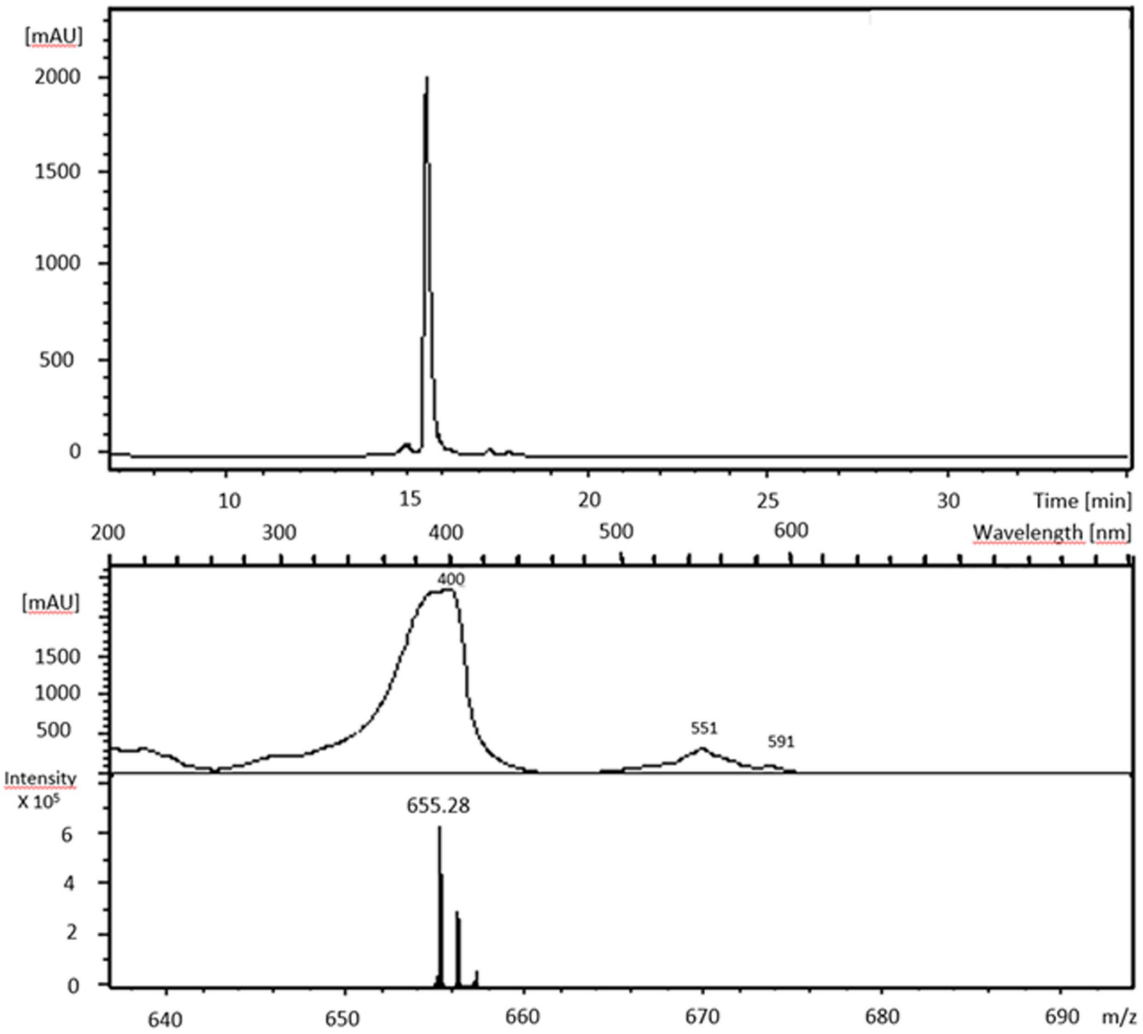
Mass spectrometric analysis and absorbance spectrum of the CgoN (YtpQ) product. Standard assays containing 1 μM freshly purified CgoN, 5 μM menadione, 3 μM FAD and 10 μM freshly prepared coproporphyrinogen III were at 30°C and 200 rpm for 120 min. Samples were analyzed after protein elimination via HPLC-MS. Shown are the HPLC run followed at 400 nm absorbance **(upper panel)**, the spectroscopic analysis of the fraction causing the major absorbance peak in the upper panel **(middle panel)** and the corresponding mass spectrum. The product is coproporphyrin III with a typical absorbance maximum at 400 nm with side peaks between 480 nm and 600 nm and a molecular mass (m/z) of 655.

**Figure 5:**
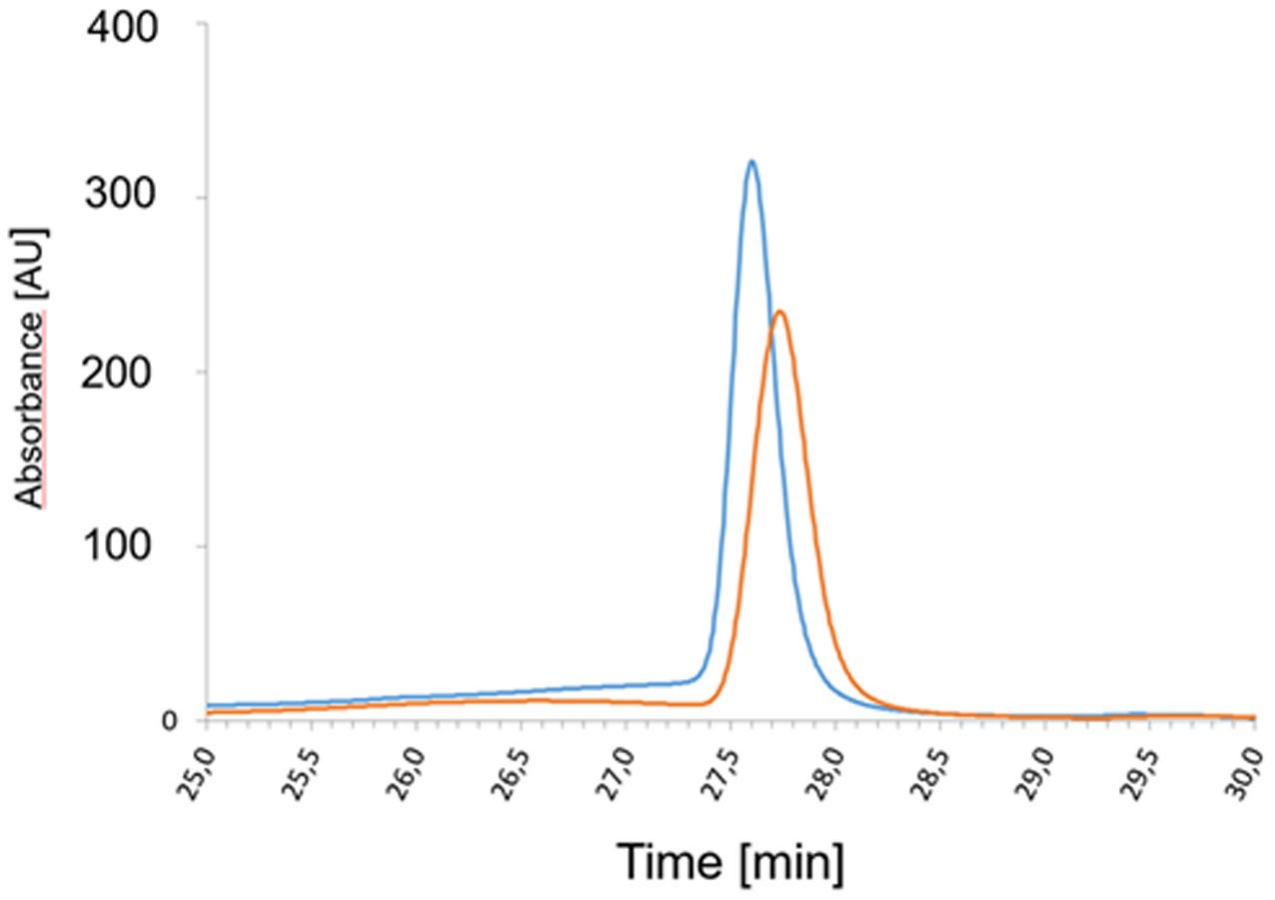
Conversion of YtpQ (CgoN)- derived coproporphyrin III by *P. megaterium cf.* into iron-coporporphyrin III (coproheme). In the first 300 μL assay 1 μM freshly purified YtpQ (CgoN), 5 μM menadione, 3 μM FAD, 10 μM freshly prepared coproporphyrinogen III were incubated at 30°C and 200 rpm for 120 min under anaerobic conditions (blue line). The second assay was composed identical to assay 1, but 1 μM freshly purified CpfC (HemH) and 10 μM (NH_4_)_2_Fe(SO_4_)_2_ were added additionally (red line). Both samples were HPLC-separated on a C_18_ column and the elution was followed by absorbance measurements at 400 nm. The column was calibrated with coproporphyrinogen III (27, 60 min) and Fe-coproporphyrinogen III (coproheme, 27, 75 min). The results of both experiments were combined in this figure. The addition of CpfC (HemH) and (NH_4_)_2_Fe(SO_4_)_2_ to YtpQ (CgoN) standard assay resulted in the formation of Fe-coproporphyrinogen III (coproheme).

The reaction product of the anaerobically YtpQ-catalyzed reaction using coproporphyrinogen III as the substrate was further characterized by high-performance liquid chromatography-mass spectrometry and absorbance spectroscopy of the same sample (Figure 4). The HPLC analysis revealed a predominant single peak (Figure 4, upper panel) with a m/z of 655 (lower panel), consistent with the presence of the anticipated reaction product, coproporphyrin III. Additionally, the absorption spectrum displayed a prominent absorption spectrum between 390 nm and 405 nm, along with minor peaks between 480 nm and 600 nm (Figure 4, middle panel), confirming the product’s identity as coproporphyrin III. Finally, we investigated whether the YtpQ-generated coproporphyrin III could be converted by the subsequent enzyme in the pathway, coproporphyrin III ferrochelatase (CpfC), into iron-coproporhyrin III (coproheme). For this purpose, the *P. megaterium hemH* gene was cloned, expressed and the resulting recombinant CpfC protein was purified via affinity chromatography (Supplementary Figure S1). This was achieved by adding 1 μM purified *P. megaterium* CpfC and 10 μM (NH_4_)_2_Fe(SO_4_) to the standard anaerobic assay that consisted of 1 μM YtpQ, 3 μM FAD, and 5 μM menadione. After incubation the reaction products were separated by HPLC and detected by absorbance spectroscopy at 400 nm. The HPLC column was previously calibrated with coproporphyrin III (27.6 min) and coproheme (27.75 min). In the absence of added CpfC, the main product eluted at 27.6 min (coproporphyrin III). In contrast, the addition of CpfC resulted in a product that eluted at 27.7 min (coproheme) (Figure 5). Notably, the latter sample displayed, no residual coproporphyrin III, indicating an almost complete conversion of the YtpQ reaction product into coproheme.

In summary, purified YtpQ, under anaerobic conditions and in a FAD-and menadione-dependent manner, converts coproporphyrinogen III into coproporphyrin III. The latter serves as substrate for the subsequent pathway enzyme CpfC. Consequently, YtpQ functions as an oxygen-independent coproporphyrinogen III oxidase in the heme biosynthetic pathway of selected Gram-positive bacteria. On this basis YtpQ should be re-named as CgoN.

### Oxygen-dependent catalysis of CgoN

Finally, the activity of YtpQ (CgoN) in the presence of oxygen (air) was investigated (Table 1). As anticipated, in the presence of oxygen and in the absence of YtpQ (CgoN), there was an elevated autooxidation of coproporphyrinogen III compared to anaerobic conditions (13 ± 3 AU). Interestingly, in the presence of YtpQ (CgoN) and FAD an enzymatic reaction using oxygen as an electron acceptor (50 ± 5 AU) was evident. The additional presence of menadione further significantly increased the activity to 141 ± 19 AU. It appears that menadione is a more efficient electron acceptor than oxygen for the enzyme.

### Structural and functional predictions for CgoN

**Figure 6:**
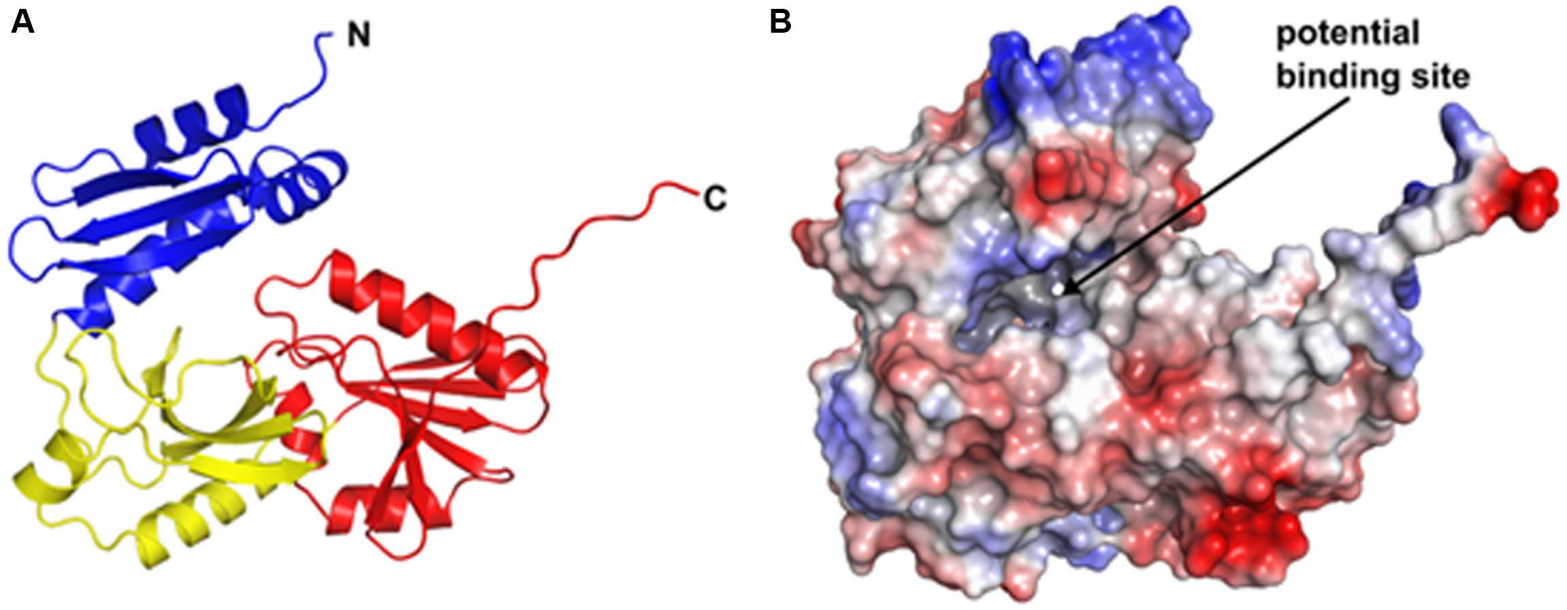
Predicted structure of YtpQ from *Priesta (Bacillus) megaterium* DSM 319. **(A)** AlphaFold2 model obtained from the AlphaFold DB (entry D5DN20). The protein consists of 270 amino acids that fold into three domains (N-terminal domain: residues 1–78, blue; central domain: residues 79–161, yellow; C-terminal domain: residues 162–270, red). **(B)** Electrostatic potential mapped to the surface of the model shown in **(A)**. Note the formation of a potential binding site at the interface between the N-terminal and central with C-terminal domains. Positive (blue) and negative (red) potential are displayed at ±100 K_b_T/e_c_. The electrostatic potential has been calculated with the APBS (Jurrus et al., 2018) plugin in PyMOL (Schiffrin et al., 2020).

To gain initial insights into structural determinants of YtpQ (CgoN), we utilized the AlphaFold2 model accessible at the AlphaFold Protein Structure Database (entry D5DN20) (Jumper et al., 2021; Varadi et al., 2022). The structure, which is modelled with a high pLDDT confidence score throughout (Figure 6 and Supplementary Figure S2), adopts a compact triangular shape and comprises three domains. Notably, the N-terminal domain may be flexible with respect to the other two as indicated by the predicted alignment error (Supplementary Figure S2). This implies a hinge-like movement of the N-terminal domain during the catalytic cycle. This notion is further supported by the remodeling of YtpQ (CgoN) with ColabFold (Mirdita et al., 2022), which positions the N-terminal domain at slightly different positions in various runs (Supplementary Figure S2). Moreover, AlphaFold models of CgoN homologues from other bacteria display the N-terminal domain at varying positions with respect to the other two domains (Supplementary Figure S3).

The movement of the N-terminal domain may create a binding site for FAD and the coproporphyrinogen III substrate (Supplementary Figure S3), while the three domains themselves do not contain cavities that could accommodate these ligands. Although structure similarity searches with DALI and Foldseek (Holm, 2022; van Kempen et al., 2023) did not identify experimentally determined protein structures with a comparable domain architecture, individual searches with the three domains of the YtpQ (CgoN) AlphaFold2 model revealed several PDB entries containing similar structural building blocks. However, the sequence identities with respect to YtpQ (CgoN) were, on average, below 15% (Supplementary Figure S3) making it challenging to infer molecular function based on these similarities.

## Discussion

In the process of extracting six electrons from either coproporphyrinogen III or protoporphyrinogen IX during heme biosynthesis, nature has evolved two distinct biological strategies to manage the abstracted electrons. The more likely ancient mechanism involves channeling these electrons into the cellular electron transfer chains for energy generation, a strategy employed by PgdH1 (HemG) and evidently also by CgoN (YtpQ) (Boynton et al., 2009; Möbius et al., 2010). However, direct coupling with electron transfer enzymes has only been demonstrated for PdgH1. As with PgdH1 (HemG), CgoN (YtpQ) also interacts with menadione (Boynton et al., 2009; Möbius et al., 2010). The redox potential of menadione (provitamin K3) with an E^0^’of − 205 mV suggests that CgoN can engage in redox-coupling with multiple anaerobic (e.g., nitrate reduction) and aerobic electron transfer chains in certain Gram-positive bacteria. An additional energetically favorable outcome of this biochemical pathway is the generation of a proton/sodium ion gradient, contributing to ATP generation.

Thus, CgoN (YtpQ) can be considered, strictly speaking, not solely an anaerobic enzyme but rather an oxygen-independent biocatalyst. Despite this, the enzyme demonstrates the ability to function directly with oxygen as an electron acceptor. Intriguingly, the presence of menadione outcompetes oxygen, suggesting that electron channeling is the favored and natural mode of action. The direct utilization of oxygen as an electron acceptor represents the second, more recent biochemical strategy employed by HemY-type enzymes (CgoX, PgoX), proving to be a more efficient way of disposing of electrons. For the oxygen-dependent CgoX a *K_M_* value of 0.31 μmol/L and a *kcat* value of 1.33 min^−1^ were reported (Lobo et al., 2009). A direct comparison reveals a 10-fold higher *K_M_* and a lower *k_cat_* for CgoN (YtpQ) with respect to the substrate coproporphyrin III (*K_M_* value of 3.95 μmol/L and a *k_cat_* of 0.63 min^−1^). Clearly, oxygen, with a redox potential of E^0^’of + 820 mV, serves as a potent electron acceptor.

Differences in the kinetics may reflect the *in vitro* test situation where an artificial (menadione) instead of natural electron acceptor (electron transport chain) is used. Additionally, H_2_O_2_, on the surface appears to be an unfavorable side-product of the oxygen-dependent pathway. However, in certain Gram-positive bacteria, it can be directly consumed by the last enzyme of the pathway (Figure 1), the coproheme carboxylase ChdC (Streit et al., 2017, 2018). This process would likely necessitate metabolic channeling through mega-complex formation of various heme biosynthetic enzymes, as observed for glutamyl-tRNA reductase (GltR) and glutamate-2,1-semialdehyde aminomutase (GsaM), glutamyl-tRNA synthetase (GltX) and GltR, and for PgoX and ferrochelatase previously (Jahn, 1992; Lüer et al., 2005; Masoumi et al., 2008).

All of these enzymes (CgoX, PgoX, PgdH1, CgoN) share a common flavin cofactor (FAD, FMN) facilitating rapid two-electron transfer. However, the protein frameworks that house these functionally-similar vary significantly among the different enzymes. HemY-type enzymes (CgoX, PgoX) belong to a FAD-containing superfamily that includes enzymes such as monamine oxidases and phytoene desaturase (Dailey and Dailey, 1998). PgdH1 (HemG) is an FMN-containing flavodoxin-type protein, likely derived from a classical electron transport protein of the flavodoxin class (Möbius et al., 2010). In contrast, YtpQ (CgoN) lacks significant amino acid sequence homology with any other class of protein. This is supported by the AlphaFold 2.0-modelled *P. megaterium* YtpQ (CgoN) protein, which failed to show any similarity with other structures of on the PDB database. Only searches using the three individual domains of the CgoN found PDB entries that contained similar structural building blocks, although in these cases the amino acid sequence identities were below 15% with respect to CgoN (Supplementary Figure S3). Consequently, YtpQ (CgoN) appears to have a unique structure unrelated to other protein families.

In agreement with its unique structure, the YtpQ (CgoN) protein is almost exclusively (98%) found in *Bacillaceae.* Almost no other *Firmicutes* harbors the enzyme, with only a few Gram-positive Actinobacteria strains also containing the protein. Interestingly, a very few single strains of well-known Gram-negative bacteria, despite the genome sequencing of many thousands of different strains, also carry the corresponding *ytpQ* gene. Since, some of them also contained *hemF* and *hemN*, this may represent a recent horizontal gene transfer event. Nevertheless, YtpQ (CgoN) is a structurally unique protein with a very narrow bacterial host range. Consequently, it seems very likely that the three different flavin-dependent enzymes, responsible for the six-electron abstraction of porphyrinogens into porphyrins, are the result of three independent evolutionary developments.

**Figure 7:**
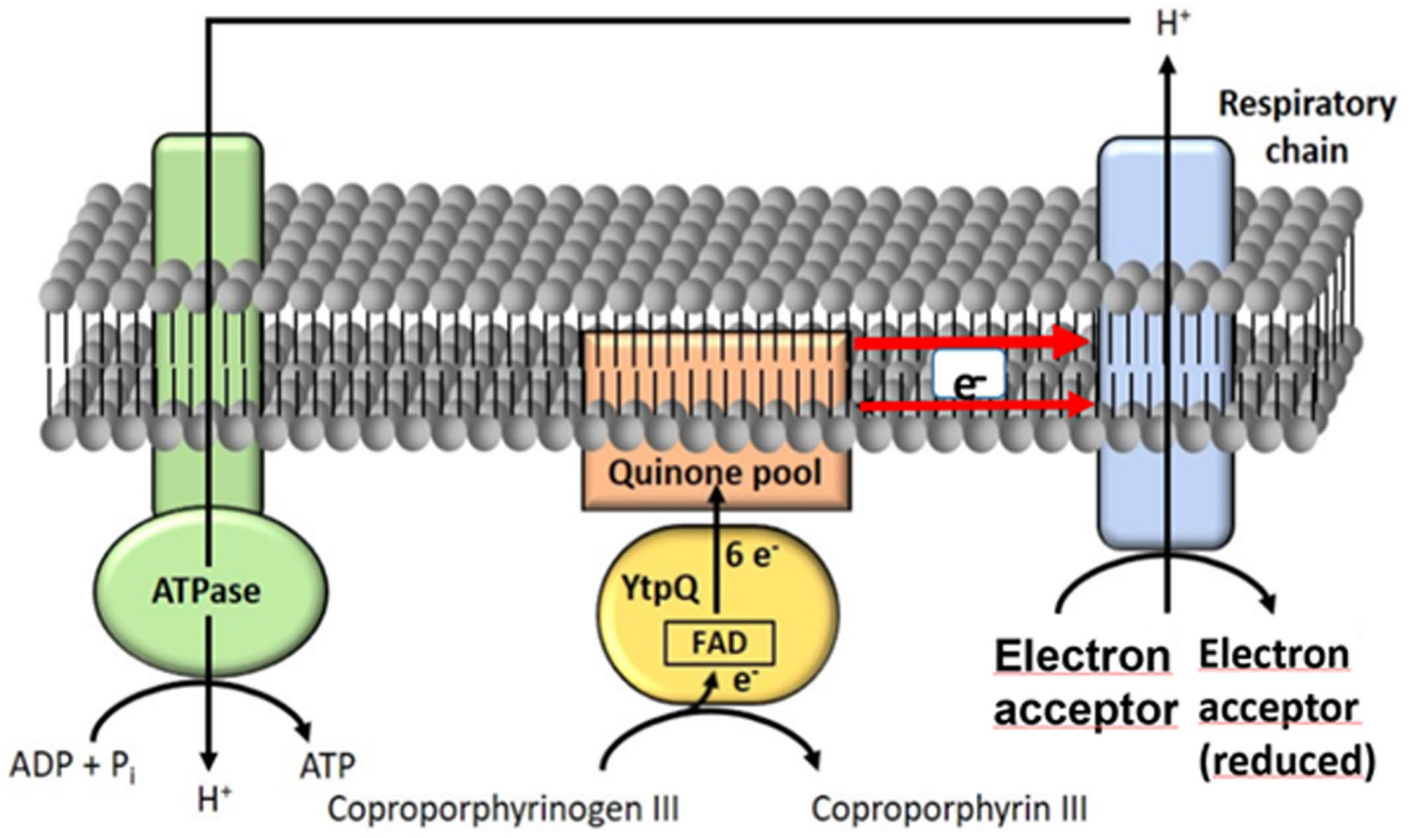
Model of YtpQ (CgoN) activity. YtpQ (CgoN) catalyzes the oxidation of coproporphyrinogen III to coproporphyrin III transferring 6 electrons via its cofactor FAD to the quinone pool. Those electrons are further channeled into the redox systems of the various aerobic and anaerobic respiratory chains (simplified depiction as light blue square). Corresponding redox reactions cause the formation of a proton (alternatively sodium ion) gradient across the membrane that in turn fuels ATP synthesis via ATPase.

Overall, based on the data provided in this study and the current literature, we have formulated the following model for CgoN (YtpQ) activity (Figure 7). CgoN (YtpQ) catalyzes the oxidation of coproporphyrinogen III to coproporphyrin III by transferring six electrons via its cofactor, FAD, to the quinone pool of the *Bacillaceae*. Subsequently, the electrons are channeled into the redox systems of the local aerobic or anaerobic respiratory chains. These redox reactions lead to the formation of a proton/sodium ion gradient across the membrane, which, in turn, drives ATP synthesis through the function of the ATPase. Much like to PgdH1 (HemG) catalysis, the energy-intensive biosynthesis of heme is sustained by the parallel generation of ATP facilitated by CgoN (YtpQ).

## Data availability statement

The original contributions presented in the study are included in the article/Supplementary material, further inquiries can be directed to the corresponding author.

## Author contributions

TM: Conceptualization, Data curation, Funding acquisition, Project administration, Supervision, Validation, Visualization, Writing – original draft. SB: Data curation, Investigation, Methodology, Visualization, Writing – original draft. VM: Data curation, Formal analysis, Investigation, Methodology, Writing – original draft. JB-dA: Data curation, Formal analysis, Visualization, Writing – original draft. RB: Supervision, Writing – original draft. AC: Data curation, Formal analysis, Methodology, Writing – original draft. TD: Data curation, Formal analysis, Writing – original draft. SG: Data curation, Formal analysis, Investigation, Methodology, Writing – original draft. WB: Formal analysis, Software, Validation, Writing – original draft. HD: Conceptualization, Investigation, Methodology, Writing – original draft. MW: Data curation, Formal analysis, Investigation, Methodology, Writing – original draft, Writing – review & editing. MJ: Conceptualization, Data curation, Funding acquisition, Investigation, Project administration, Visualization, Writing – original draft, Writing – review & editing. DJ: Conceptualization, Data curation, Funding acquisition, Project administration, Resources, Supervision, Writing – original draft, Writing – review & editing.

## Footnotes

^1^http://www.jcat.de/
