# Supplementary material for: The alternative coproporphyrinogen III oxidase (CgoN) catalyzes the oxygen-independent conversion of coproporphyrinogen III into coproporphyrin III
Source: Front Microbiol. 2024 Mar 13;15:1378989. doi: 10.3389/fmicb.2024.1378989 (PMC10965808; doi:10.3389/fmicb.2024.1378989)
Supplement: Supplementary file 1 [file Data_Sheet_1.docx]

**Supplementary Material**

**The Alternative Coproporphyringen III Oxidase (CgoN) Catalyzes the Oxygen-independent Conversion of Coproporphyrinogen III into Coproporphyrin III**

**Toni Mingers^1,2^, Stefan Barthels^1^, Violetta Mass^1^, José Manuel Borrero-de Acuña^8^, Rebekka Biedendieck^,6^, Ana Cooke^3,7^, Tamara A. Dailey^4^, Svetlana Gerdes^9^, Wulf Blankenfeldt^5^, Harry A. Dailey^4^, Martin Warren^3^, Martina Jahn^1^, and Dieter Jahn^1,6,^***

^1^ Institute of Microbiology, Braunschweig University of Technology, 38106 Braunschweig, Germany, [m.jahn@tu-bs.de](mailto:m.jahn@tu-bs.de), [s.barthels@tu-bs.de](mailto:s.barthels@tu-bs.de), [violettam26.vm@gmail.com](mailto:violettam26.vm@gmail.com),

^2^ Pieris Pharmaceuticals GmbH, Zeppelinstr. 3, 85399 Hallbergmoos, Germany, [toni.mingers@gmail.com](mailto:toni.mingers@gmail.com),

^3^ School of Biosciences, University of Kent, Canterbury, Kent, United Kingdom; Quadram Institute Bioscience, Norwich Research Park, Rosalind Franklin Road, Norwich Research Park, Norwich, NR4 7UQ, United Kingdom, [Martin.Warren@quadram.ac.uk](mailto:Martin.Warren@quadram.ac.uk)

^4^ Department of Microbiology, University of Georgia, Athens, Georgia, USA, [hdailey@uga.edu](mailto:hdailey@uga.edu)

^5^ Department of Structure and Function of Proteins (SFPR) Helmholtz Centre for Infection Research (HZI), 38124 Braunschweig, Germany, and Institute for Biochemistry, Biotechnology and Bioinformatics, Braunschweig University of Technology, 38106 Braunschweig, Germany, [wulf.blankenfeldt@helmholtz-hzi.de](mailto:wulf.blankenfeldt@helmholtz-hzi.de)

^6^ Braunschweig Integrated Center of Systems Biology, Braunschweig University of Technology, 38106 Braunschweig, Germany, [d.jahn@tu-bs.de](mailto:d.jahn@tu-bs.de), r.biedendieck@tu-bs.de

^7^Syngenta UK Ltd., Cambridge, Capital Park, CPC4, cb215xe , United Kingdom, [Ana.Cooke@syngenta.com](mailto:Ana.Cooke@syngenta.com)

^8^Departamento de Microbiología, Facultad de Biología, Universidad de Sevilla, Sevilla, Spain, [jbdeacuna@us.es](mailto:jbdeacuna@us.es)

9Svetalana Gerdes9, Dupont Daniscao Research Center, 200 Powder Mill Road,Wilmington, DE 19803, [gerdessvetlana@gmail.com](mailto:gerdessvetlana@gmail.com)

***** Correspondence: e-mail: [d.jahn@tu-bs.de](mailto:d.jahn@tu-bs.de), Tel.: +49 531-391-55101

**FIGURE LEGENDS**

**FIGURE S1. SDS gel of the production and purification of *P. megaterium* CpfC (HemH).** Recombinant *P. megaterium* CpfC (HemH) was produced in *E. coli* BL 21 as outlined in Material and Methods. The image shows an InstantBlue™ stained 12 % SDS polyacrylamide gel after electrophoresis. Pierce™ unstained molecular weight marker protein ranging M_r_ from 25,000 to 116,000 are shown in lane M. Further, the protein composition of the flow through of the glutathione agarose (lane 1), washing fraction 1 (lane 2); washing fraction 5 (lane 3) and elution fraction 1 (lane 4) after PreScission™ protease cleavage are shown. Fraction 4 contains a major CpfC (HemH) band and residual amounts of the CpfC-GST fusion protein and GST.

**FIGURE S2.** Details of the AlphaFold2 model of YptQ from *Priesta (Bacillus) megaterium* DSM 319 obtained from the AlphaFold DB (entry D5DN20). **(A)** The structure has been colored according to the pLDDT value calculated by AlphaFold2. Note the high confidence score of the core structure. **(B)** Matrix plot of the predicted alignment error (PAE) shows that the position of the N-terminal domain with respect to the other two domains has higher uncertainty, suggesting that this domain is flexible with respect to the rest of the structure.

**FIGURE S3.** Movement of the N-terminal domain as indicated by repetitive AlphaFold2-modeling. Five models of YptQ from *P. megaterium* DSM 319 (thin lines in different shades of grey) were calculated with ColabFold and then superimposed onto the model obtained from AlphaFold DB. Note that the N-terminal domain finds different positions with respect to the remainder of the structure in these models.

**FIGURE S4.** Movement of the N-terminal domain as indicated by comparison to AlphaFold DB models of homologous proteins identified with FoldSeek. The model of YptQ from *P. megaterium* DSM 319 obtained from AlphaFold DB (entry D5DN20) is shown in thin black lines. Structure Q2FXI7 is an uncharacterized protein from *Staphylococcus aureus* (strain NCTC 8325 / PS 47) that shares 54% sequence identity to YptQ from *P.* *megaterium* DSM 319; K0EXG2 is an uncharacterized protein from *Nocardia brasiliensis* ATCC 700358 (sequence identity 12%). Note the different positions of the N-terminal domain with respect to *P. megaterium* YptQ.
