# Supplementary figures and images for: The alternative coproporphyrinogen III oxidase (CgoN) catalyzes the oxygen-independent conversion of coproporphyrinogen III into coproporphyrin III
Source: Front Microbiol. 2024 Mar 13;15:1378989. doi: 10.3389/fmicb.2024.1378989 (PMC10965808; doi:10.3389/fmicb.2024.1378989)

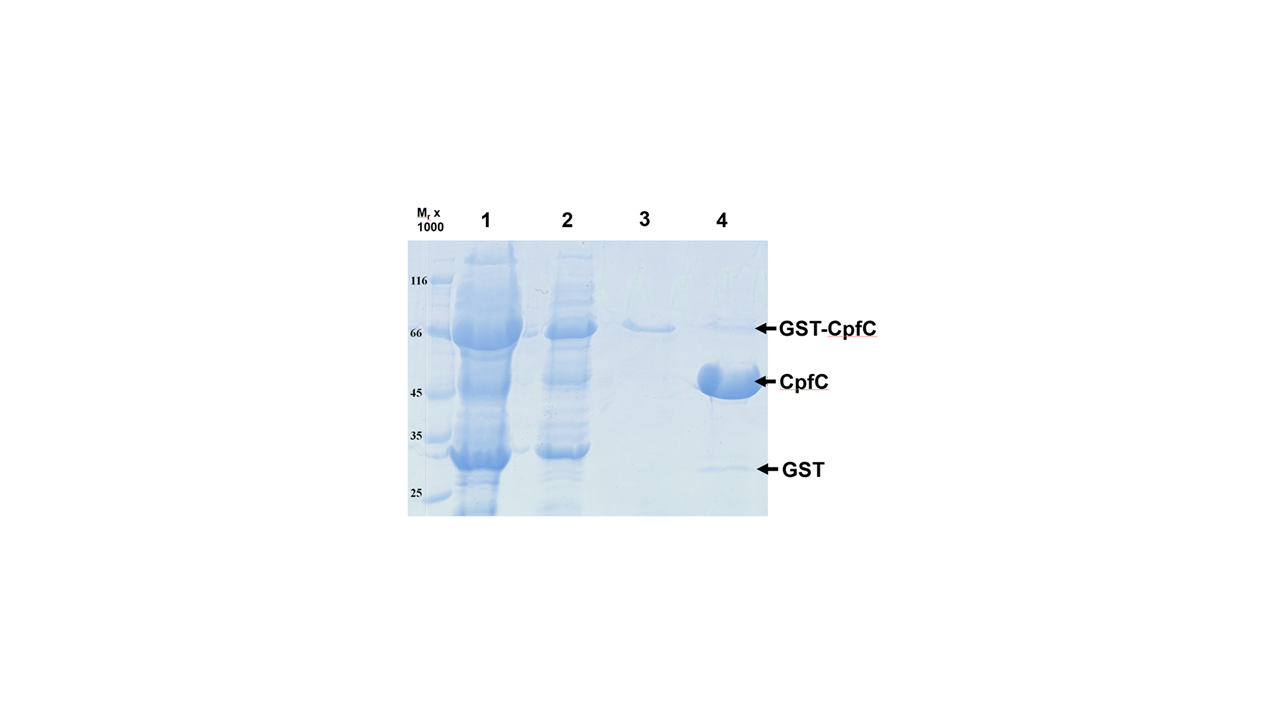

Supplement: Supplementary file 2 [file Image_1.tif]

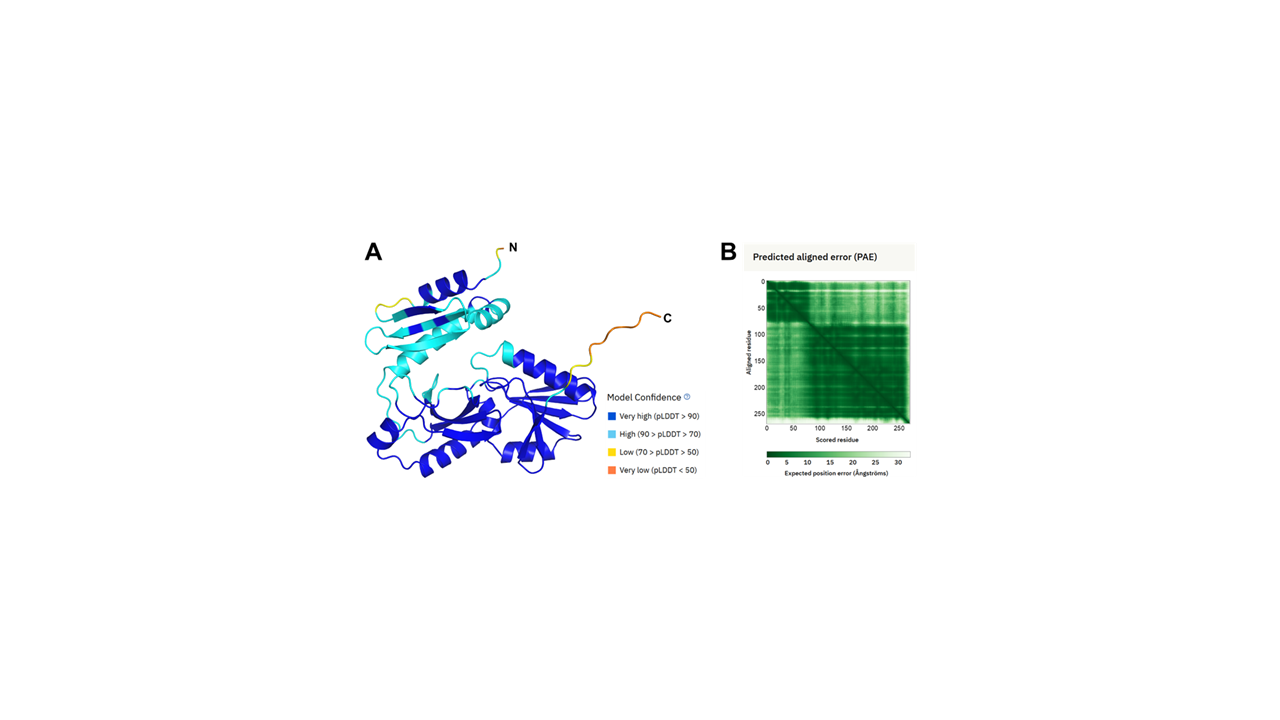

Supplement: Supplementary file 3 [file Image_2.tif]

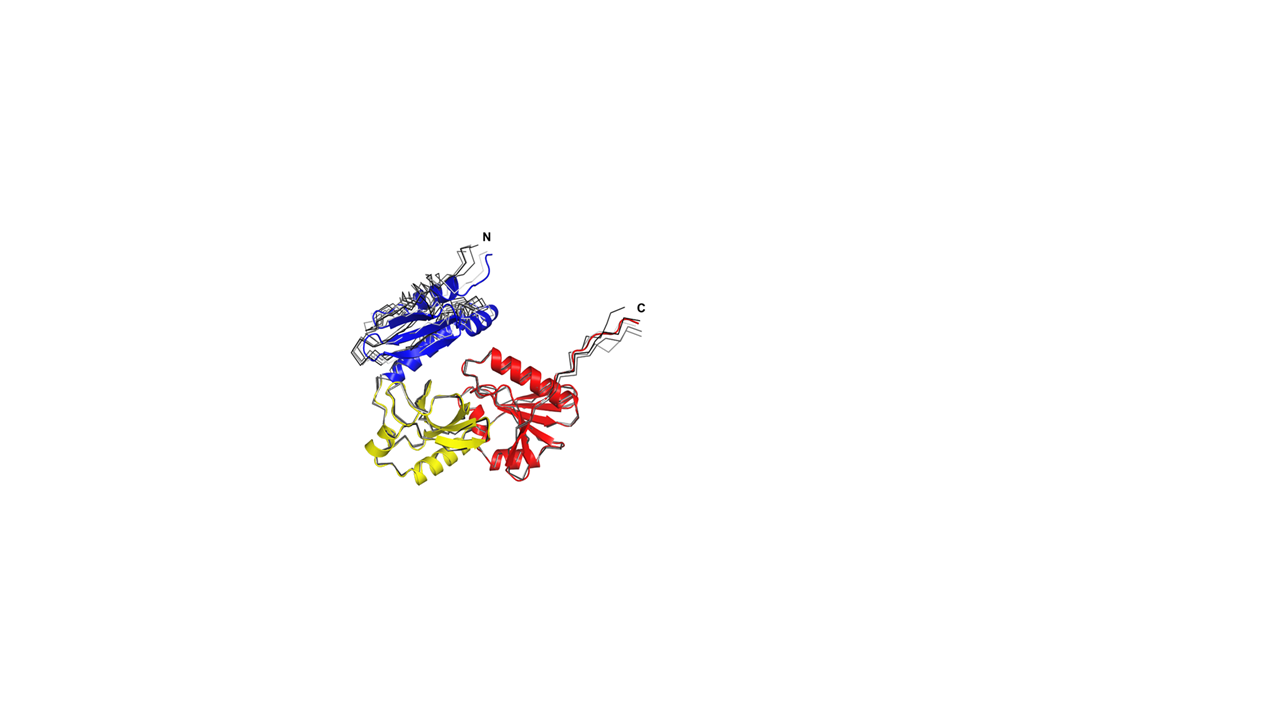

Supplement: Supplementary file 4 [file Image_3.tif]

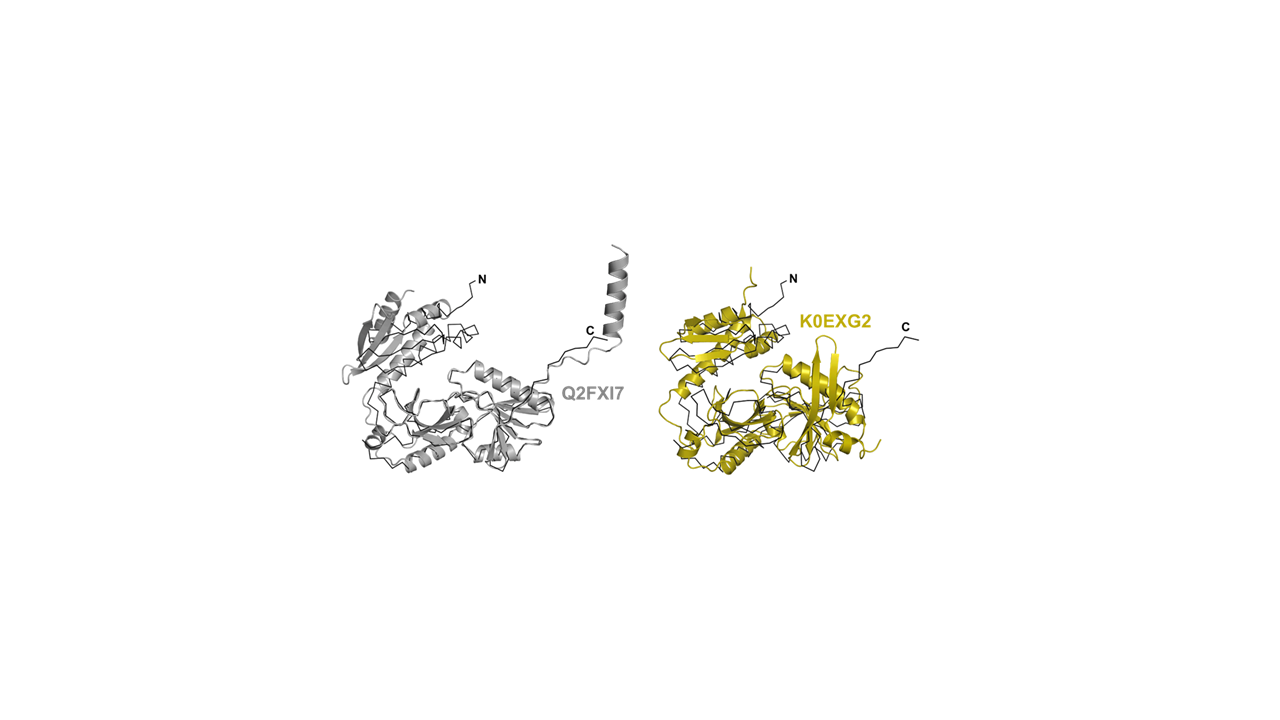

Supplement: Supplementary file 5 [file Image_4.tif]
